# A Phase I Clinical Trial to Assess Safety and Tolerability of Injectable Collagenase in Women with Symptomatic Uterine Fibroids

**DOI:** 10.1007/s43032-021-00573-8

**Published:** 2021-04-29

**Authors:** Bhuchitra Singh, Holly Sims, Irene Trueheart, Khara Simpson, Karen C. Wang, Kristin Patzkowsky, Thomas Wegman, Jean-Marie Soma, Rosina Dixon, Friederike Jayes, Kristin Voegltine, Gayane Yenokyan, Szu-Chi Su, Phyllis Leppert, James H. Segars

**Affiliations:** 1 Department of Gynecology and Obstetrics, Division of Reproductive Sciences & Women’s Health Research, Johns Hopkins University School of Medicine, 720 Rutland Avenue, Ross Research Building, Room 624, Baltimore, MD 21205 USA; 2 Department of Gynecology and Obstetrics, Minimally Invasive Gynecologic Surgeons, Johns Hopkins University School of Medicine, Baltimore, MD USA; 3 BioSpecifics Technologies Corporation, Lynbrook, NY USA; 4 Department of Obstetrics and Gynecology, Duke University School of Medicine, Durham, NC USA; 5 Biostatistics, Epidemiology and Data Management Core, Johns Hopkins School of Medicine, Baltimore, MD USA; 6 Johns Hopkins School of Public Health, Biostatistics Center, Baltimore, MD USA

**Keywords:** Uterine fibroids, Collagenase, Phase 1 study, *Clostridium histolyticum*, Leiomyoma

## Abstract

Uterine fibroids feature excessive deposition of types I and III collagen. Previous ex vivo studies showed an FDA-approved collagenase (EN3835)-digested types I and III collagen fibers in fibroid tissues; however, collagenase had not been evaluated in vivo for effects on uterine fibroids. The objective was to assess the safety and tolerability of collagenase injection directly into uterine fibroids. This was a prospective, open label, dose escalation study. The study participants were fifteen women aged 35–50 years with symptomatic uterine fibroids planning to undergo hysterectomy. Three subjects received saline and methylene blue, three subjects received a fixed dose of EN3835, and 9 subjects received stepped, increasing dosages of EN3835, all by transvaginal, ultrasound-guided injections. Primary outcome measures were safety and tolerability of the injection and change in collagen content between treated and control tissues. There were no significant adverse events following injection of EN3835 into uterine fibroids. Masson’s trichrome stains revealed a 39% reduction in collagen content in treated samples compared to controls (*p* <0.05). Second harmonic generation (SHG) analysis showed treated samples to have a 21% reduction in density of collagen compared to controls. Picrosirius-stained collagenase-treated fibroids showed collagen fibers to be shorter and less dense compared to controls. Subjects reported a decrease in fibroid-related pain on the McGill Pain Questionnaire after study drug injection in Group 2 at both 4–8 days and 60–90 days post-injection. The findings indicated that injection of collagenase was safe and well tolerated. These results support further clinical investigation of collagenase as a minimally invasive treatment of uterine fibroids. NCT0289848.

**Supplementary Information:**

The online version contains supplementary material available at 10.1007/s43032-021-00573-8.

## Introduction

Uterine leiomyomas or fibroids are the most common benign tumors of the female reproductive system and pose a significant problem for millions of women [1]. By age 50, uterine fibroids are diagnosed in more than 80% of African American and 70% of Caucasian women [2]. The estimated direct annual costs of medical and surgical management for fibroids range from approximately 4 to 9 billion USD [3].

Fibroids arise within smooth muscle cells. However, multiple studies show that the bulk of these tumors is composed of an extracellular matrix (ECM) mostly consisting of disorganized, altered, highly cross-linked collagen fibers [4–8]. The ECM component of the fibroid has a direct effect on tumor growth by induction of fibrosis that leads to a decreased rate of apoptosis and increased collagen deposition [9, 10]. In a recent study, untreated fibroids demonstrated collagen-rich fibrosis ranging from 37 to 77%. After ex vivo treatment with collagenase for 96 h, fibrosis ranged from 2.4 to 5.3% [11]. The reduction was associated with a decrease in tissue stiffness and loss of collagen fibers in treated fibroids as compared to control tissues [11, 12]. Specifically, EN3835 digests types I and III collagens which are abundant in fibroids [10, 13]. We postulated that by digesting the collagen of fibroids, the subsequent debulking of the tumor may result in reduced fibroid symptoms such as pain or bleeding [12].

Evidence from minimally invasive therapies currently available for uterine fibroids, such as uterine artery embolization or uterine fibroid ablation using MR-guided focused ultrasound, supports the tenet that reduction in fibroid size can translate into a reduction in fibroid-related symptoms [14–17]. EN3835 is a non-hormonal treatment, possibly affording patients a new minimally invasive option for fibroid treatment.

As a therapy, purified collagenase *Clostridium histolyticum (EN3835)* was FDA-approved for the treatment of Dupuytren’s contracture by local injection in 2010 and for Peyronie’s disease in 2013 [18–20]. EN3835 consists of collagenases of classes I and II with a potent binding affinity to interstitial collagens, especially collagens I and III. Class I EN3835 has an especially high affinity to mature triple helical interstitial collagen at a preferred cleavage site in the N and C termini. Class II EN3835 cleaves the inner peptides, and its preferred substratum is small denatured peptides [21–26]. The extracellular matrix in a fibroid is abundantly composed of collagens types I, III, and V, making fibroids a logical target for EN3835 [9]. Notably, EN3835 does not degrade the type IV collagen found in the basement membranes of the nerves and blood vessels [18, 19]. This is important as fibroids can be vascularized and are surrounded by a neurovascular pseudocapsule. Furthermore, EN3835 is inhibited by serum proteins and is rapidly degraded in the circulation [19, 27, 28]. These features were confirmed in clinical trials for Peyronie’s disease. After treatment, antibodies directed against EN3835 I and II were detected in serum; however, no adverse effects were noted as a result [18, 19].

The aim of this clinical trial was to explore the safety and tolerability of using collagenase *Clostridium histolyticum* (EN3835) in women with symptomatic uterine fibroids. We hypothesized that treatment of clinically relevant leiomyomas with collagenase EN3835 would be feasible and reduce the collagen content of the fibroids.

## Materials and Methods

### Study Design

This was an open-label, dose-escalation study of EN3835 in women with symptomatic uterine fibroids undergoing hysterectomy at Johns Hopkins Hospital, Baltimore, MD, USA. The Institutional Review Board at Johns Hopkins School of Medicine approved the study protocol and all procedures (IRB00091412). The clinical trial was registered with clinicaltrials.gov (NCT02889848). All study drug injections were performed at the Johns Hopkins Outpatient Ambulatory Surgery Center. This was a pilot study with a sample size of 15 subjects based on pre-clinical results of effective dosages and incubation times. This was a pilot study, not a statistically powered study. Each subject had one fibroid injected with EN3835. This study was conducted in accordance with US and international standards of Good Clinical Practice (FDA Title 21 CFR part 312 and International Conference on Harmonization guidelines), applicable government regulations, and institutional research policies and procedures.

**Fig. 1:**
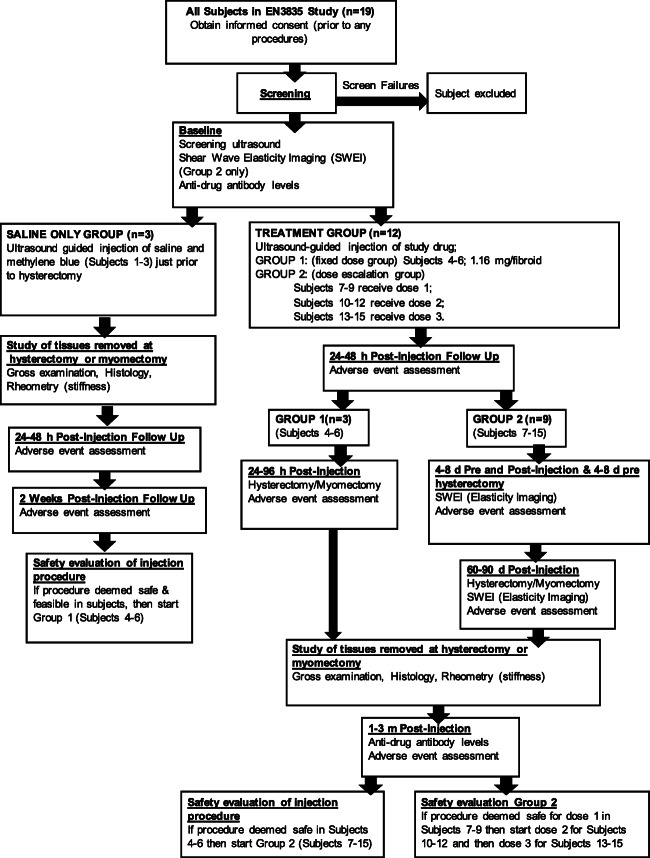
Study design. Detailed structure of the study activities. Standard clinical care was provided pre- and post- hysterectomy. M=month, SWEI=shear wave elastography index

The safety and tolerability of EN3835 were evaluated using a stepwise approach for the administration of the study drug (Fig. 1). Saline-only subjects were treated first, followed by Group 1 fixed dose subjects, and then Group 2 subjects (progressing from lowest dosage group to highest dosage group). The administration of study drug in each dosage group was predicated on demonstration of safety and tolerability in prior dosage group. A Safety Committee was established to assess and review the safety of the study. The three subjects in the saline-only Group (*n*=3) were injected with normal saline and methylene blue, immediately prior to their hysterectomy. This served as the feasibility group for the injection procedure and drug delivery. Group 1 (*n*=3) was the fixed dose group; all three subjects received 1.16 mg of the study drug 24–48 h prior to hysterectomy. This dose was selected based on previously approved dosing in Dupuytren’s disease. Group 2 (*n*=9) was further divided into three subgroups (*n*=3/ subgroup), each receiving a higher dose of the study drug than the last subgroup (1.68, 3.35, and 5.028 mg, respectively, as the maximum doses). Each subgroup included three subjects who underwent hysterectomy 60–90 days post-study drug injection.

Injected fibroids were collected post-hysterectomy, and gross examination was performed. The fibroid samples collected at hysterectomy were assessed for collagen content and distribution, percentage change of collagen content by histology stains, and apoptosis by TUNEL staining.

### Study Subjects

Recruitment occurred through referrals from gynecologists and radio advertisements. The discussion for enrollment was deferred until the women made an independent decision with their gynecologist to undergo surgical management for fibroids such as hysterectomy or myomectomy. Patients who expressed interest and qualified per study criteria signed the informed consent form to be enrolled in the study.

### Eligibility Criteria

Women aged 35–50 years old with symptomatic uterine fibroids, with at least one typical intramural fibroid with diameter 3–10 cm, who had completed childbearing and were willing to practice contraception throughout the duration of the study were included in the study. Estrogen and progesterone levels were evaluated for all subjects to confirm pre-menopausal state at the time of study enrollment. Interim hormonal treatment until hysterectomy was allowed (only one subject received hormonal treatment while being enrolled in the study). MRI was performed for all study subjects and only those with “typical” fibroids, visualized as hypo-intense on a T-2 weighted MRI scan, were selected. A screening ultrasound with Doppler was performed for all study subjects to identify the best route for the study drug injection.

Women with BMI > 40kg/m^2^, history of allergic reaction to EN3835, cancer within the past 5 years, abnormal liver function test (> 20% elevation), severe anemia (HCT < 30%), recent rapid growth of fibroids, and type 0 submucosal, pedunculated, and subserosal fibroids were excluded from this study. The subjects who met the eligibility criteria were assigned to the next available study group based upon the date of their enrollment in the study and the timing of their hysterectomy.

### Study Drug Administration

**Fig. 2:**
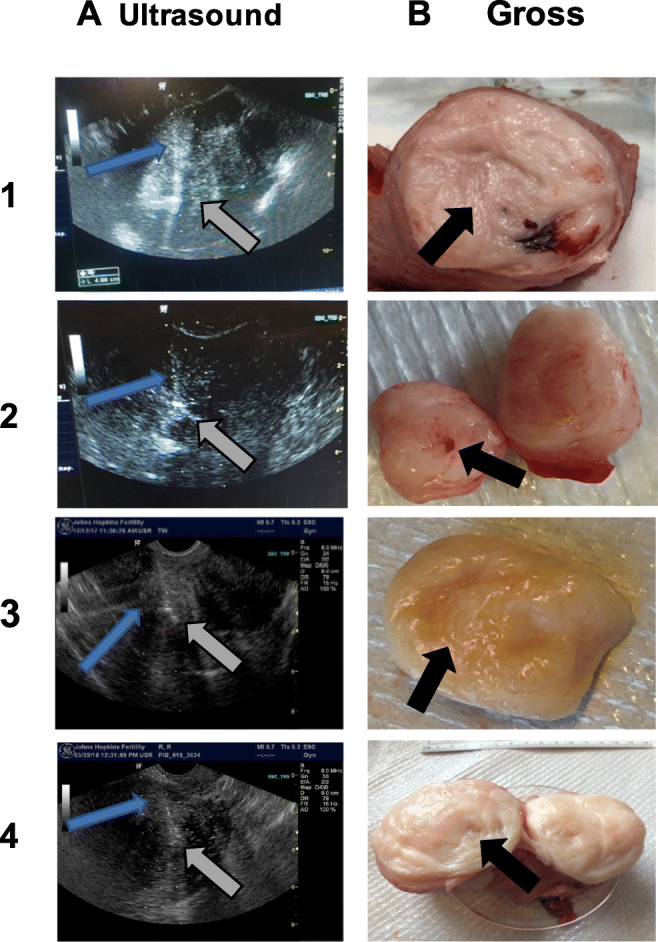
Representative images of the ultrasound guided study drug injection (Column A), gross hemi-section of the fibroid tissue (Column B) injected with various doses of collagenase group1, 1.16 mg (row 1), and group 2 dose 1, (row 2), dose 2, (row 3), and dose 3, (row 4) with 1.68, 3.35, and 5.028 mg as the maximum doses, respectively. The blue arrows mark the needle, grey arrows mark the study drug, and the black arrows mark the area of digestion by the study drug in the hemisected fibroid sample. The areas of digestion were visibly darkened and softened (black arrows, rows 1, 3, 4), and sometimes completely liquefied, as in the hole marked row 2 (black arrow)

All subjects received a single injection of either saline (saline-only group, *n*=3) or EN3835 (Groups 1 and 2, *n*=12) into one intramural fibroid. The injected control fibroid was well visualized on ultrasound examination and had a clear path for transvaginal injection. All injections were performed by a single surgeon (J.S.) who had prior experience with transvaginal insertion of a retrieval needle under ultrasound guidance for infertility procedures. For the injection, subjects were sedated, positioned in lithotomy position, and fibroids were injected. To avoid injury to blood vessels, color flow Doppler was used to identify the best route to the center of the selected fibroid. A conventional 17 G, 350 mm, conventional single lumen follicle aspiration needle (manufactured by Vitrolife) was used for the study drug injection. All injections were accurately administered within 3–5 cm of the vaginal mucosa, and all injections were visualized via ultrasound (Fig. 2). The study drug was injected into the center of the fibroid to ensure safe distribution of the study drug and for accurate assessment of collagen content change once the sample was collected post-hysterectomy. Slight repositioning of the needle was done to ensure localized infusion and delivery of the study drug. The study drug injection took on average between 1.2 to 2 minutes to complete. The entire procedure, including time to sedate and position the subject, required 20–25 min. The subjects in Groups 1 and 2 remained at Johns Hopkins for 4 h post-study drug injection to be monitored for possible immediate adverse events, including hypersensitivity reactions. All subjects were assessed at 24 h post-injection for any untoward effects.

### Study Drug Dosage

The first 3 subjects in the study received methylene blue 1% in saline in the OR immediately prior to hysterectomy. The dye was injected to confirm the injection site in the fibroid sample upon inspection of the fibroid sample post-hysterectomy. Upon completion of the saline-only group, three subjects (Group 1) received 1.16 mg of EN3835, regardless of fibroid size. Most fibroids are spherical in shape; hence, the volume of EN3835 was calculated according to the formula for volume of a sphere. Approximately 50–70 ml were injected for each 1 cm^3^ of fibroid volume, to a maximum volume of 1.676 ml/ fibroid regardless of fibroid volume. For Group 2 subjects, using an injection volume of 0.05ml/cm^3^ of fibroid volume, doses of study drug delivered per escalation group was 0.05, 0.1, and 0.2 mg/cm^3^ of the fibroid, but no subject was to receive more than 1.68, 3.35, and 5.028 mg for dose 1, 2, and 3, respectively. The maximum doses were capped at two- and threefold that of dose 1, since this was the first safety study of EN3835 injection into uterine fibroids.

### Assessments

#### Safety and Tolerability

A primary outcome of this study was to assess the safety and tolerability of EN3835 following a one-time injection directly into a uterine leiomyoma. This was evaluated by thorough follow-up of the subjects from enrollment to last follow-up visit at predetermined intervals, and all adverse events were evaluated and reported by the study team. The subject’s laboratory values and vital signs pre- and post-injection were recorded. For both Group 1 and Group 2, to determine systemic exposure to study drug, plasma samples were collected at pre-dose, and 5, 10, 30, 60, and 240 min post-injection. Serum samples were also analyzed for antibodies directed against the EN3835 preparation, anti-AUX-I and anti-AUX-II antibodies, at baseline and last follow-up visit.

#### Change in Collagen Content

A second primary outcome was reduction in collagen content as assessed by histology staining (Masson trichrome) followed by computer assisted morphometry. For each subject, the injected (treated) fibroid and one additional non-injected fibroid (control) were harvested post-hysterectomy. The samples were hemisected to expose the center of the fibroid, paraffin-embedded, and sectioned in 5-μm slices. Effects on collagen content and distribution were compared between control and treated fibroids using Masson’s trichrome and Picrosirius red stains. Second-harmonic imaging microscopy, known as Second Harmonic Generation (SHG, or frequency doubling), uses laser light to directly image a material with non-centrosymmetric protein assemblies, such as collagens, without use of exogenous labels [29]. SHG was used to compare collagen organization and distribution between control and treated fibroids.

Collagen content was quantified in Masson’s trichrome-stained slides of control and treated fibroids from each subject. ImageJ was used to obtain pixel counts representing areas of stained collagen in 9 grids with equal area in the center of each fibroid sample [30]. Treated and control fibroids were compared for each subject. However, fibroids can be heterogeneous in collagen density and stiffness, and the control fibroids may not be representative of other fibroids from the same woman [31]. Therefore, we performed an additional analysis to evaluate changes in collagen content by normalizing control values and combining all controls (*n*=12) to adjust for the biological variability of collagen content in fibroids between subjects. For this analysis, each treatment group was compared against this pooled, normalized control group of un-injected subject-matched fibroid samples. TUNEL assay was used to compare percent of apoptosis between control and treated fibroids. Tissue sections incubated with DNase I for 10 min at 15–25°C, prior to labeling solution introduction, were used as positive control, and sections incubated with label solution alone were used as negative control.

Planned secondary outcomes included the measurement and change in size of the treated fibroid, and assessment of elasticity of uterine fibroids by B shear wave elasticity imaging (SWEI) technology.

### Patient-Reported Outcomes

Subjects in Group 1 and 2 completed study-related questionnaires. Part 1 of the Uterine Fibroid Symptom Quality of Life Questionnaire (UFS-QOL) specifically evaluated severity of physical symptoms associated with fibroids, and Part 2 of the UFS-QOL evaluated health-related quality of life associated with fibroids [32–34]. The McGill Pain Questionnaire collected detailed data about the pain associated with fibroids and evaluated the impact on pain from the study drug injection [35, 36]. The visual analogue scale (VAS) for pain was used to evaluate fibroid-related pain on a 0–10 Likert scale (higher score = worse pain) [37, 38]. The questionnaires were administered at baseline and post-study drug injection to assess fibroid-related symptoms such as menorrhagia and pain. For Group 1, this was 24–48 h post-study drug injection before hysterectomy, and 2 weeks post-hysterectomy. For Group 2, questionnaires were 4–8 days post-study drug injection, and 60–90 days post-study drug injection before hysterectomy.

### Statistical Analysis

#### Safety and Tolerability

Frequency of adverse events was compared between dosage groups (including the saline-only group) at standardized intervals determined by dosage group. No statistical tests were applied due to multiplicity, the small number of subjects, and the small number of events. Paired *t*-tests were used to assess changes in lab values and vital signs between baseline and post-injection.

#### Change in Collagen Content

Changes in collagen content were assessed using linear mixed models to estimate the ratio of intensity density of collagen by treatment and control group (Stata/IC 14.0 and Excel 2013 software); all tests were performed at 0.05 level of statistical significance. The models included treatment groups and their interaction as the primary predictors.

#### Patient-Reported Outcomes

Changes in the patient reported outcomes (overall and by subject) from baseline to post-injection were quantified and described to assess trends in safety and tolerability, but assessment of significance was limited by the small sample size.

## Results

### Demographics

Of the 19 patients screened, all of whom planned on undergoing hysterectomy, 15 women who met the study’s eligibility criteria were enrolled. The average age of the study subjects was 44.7 ± 2.6 years. The ratio of black to white women was 3:2, similar to the racial prevalence of fibroids. The average BMI of the study participants was 30.48 kg/m^2^. The study team noted that average BMI was lower (24.7 kg/m^2^) for the Group 2 Dose 2 subjects. During the screening visit, a detailed medical history and concomitant medication review, physical exam with pelvic exam, and laboratory blood test were performed to ensure eligibility. The baseline characteristics of the 15 subjects are presented in Table 1.

**Table 1:** Summary of baseline characteristics of study subjects

EN3835 study subjects					
Study group	Saline only	Group 1	Group 2 Dose 1	Group 2 Dose 2	Group 2 Dose 3
Age, years, mean (SD)	46.0 (2.6)	44.0 (1.0)	46.0 (3.0)	42.0 (1.7)	45.3 (2.9)
Female, *n*, (Black: White)	3 (1:2)	3 (2:1)	3 (2:1)	3 (2:1)	3 (2:1)
Weight, kg. mean(SD)	70.2 (4.8)	105.8 (4.1)	90.3 (20.4)	59.7 (8.6)	90.5 (21.6)
Height, m, mean (SD)	1.6 (0.0)	1.7 (0.0)	1.6 (0.1)	1.6 (0.1)	1.7 (0.1)
Body Mass Index, kg/m^2^	27 (2.5)	34.8 (1.0)	33.8 (3.9)	24.7 (4.1)	32.1 (5.4)

Values are presented as mean with standard deviation (SD)

### Safety and Tolerability

No serious adverse event occurred in any subject and no adverse events led to discontinuation of a subject in the study. No allergic reactions were observed in the 12 subjects that received study drug. Eleven of 15 subjects (73.3%) experienced at least one adverse event, of which 68.18% were mild and 31.18% were moderate in severity. Four of the 30 mild adverse events were possibly treatment-emergent: vaginal discharge (1) and vaginal spotting (3) but did not require any medical intervention (Table 2). Symptoms such as pain and bleeding that are normally associated with fibroids were not recorded as adverse events unless the condition worsened or was unusual for the subject. None of the moderate adverse events required medical or surgical intervention. No subject reported an increase in either pain or bleeding related to fibroids due to the study drug injection.

**Table 2:** Summary of treatment emergent adverse events (all subjects)

	All Subjects(*n* = 15) *n* (%)
Treatment-Emergent AEs	
Mild*	30 (68.18)**
Moderate	14 (31.18)
Severe	0
Drug related	0
Serious adverse events	0
Drug-related serious adverse events	0

*Only 4 mild treatment emergent adverse events were deemed possibly related to the study drug

**No medical intervention was needed to control the 4 possibly drug related treatment emergent adverse events

There was no association between the dose of collagenase received and the number and severity of adverse events. No other safety concerns such as changes in laboratory tests or abnormal vital signs occurred throughout the duration of the study for any of the subjects.

Blood samples for pharmacokinetic studies were collected pre-dose and at 5, 10, 30, 60, and 240 min following study drug injection. None of the study subjects had a serum concentration of the drug prior to start of the injections. Plasma concentrations peaked between 7.6 and > 160 ng/ml at 5–10 min and fell to undetectable by 4 h post-injection. Anti-AUX-I and anti-AUX-II antibodies were analyzed in serum samples obtained from subjects in Groups 1 and 2 at baseline and the final study visit (3 months post-hysterectomy), and an additional sample was taken from subjects in Group 2 at 60–90 days post-study drug injection. Exposure to EN3835 resulted in a minimal increase in anti-AUX-1 and anti-AUX-II antibodies, with the highest titers present in Group 2 Dose 3.

### Gross Fibroid Examination

The targeted delivery to the center of the fibroids was determined to be feasible based on the three saline-only group subjects. Delivery of methylene blue injected transvaginally under ultrasound guidance confirmed the method (Supplemental Figure 1). In Groups 1 and 2, treated fibroid tissues were noticeably soft to palpation on gross examination. Some samples injected with higher dosages of EN3835 showed liquefaction at the area of injection (Fig. 2). The digestion of collagen did not extend beyond the pseudocapsule of any fibroid. The details of the size of the fibroids injected and the study drug dosage per fibroid are shown in Supplemental Table A.

### Collagen Density and Distribution

**Fig. 3:**
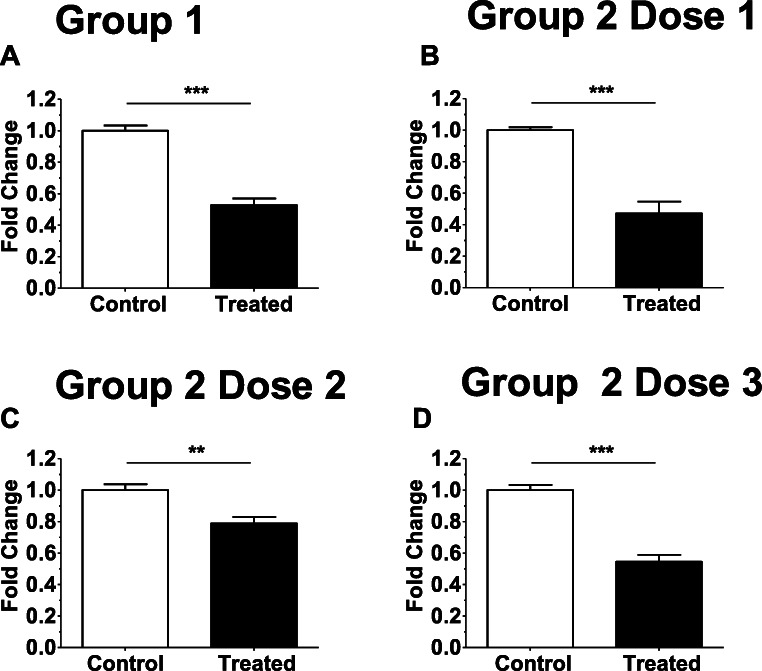
Changes in collagen content among tissues summarized for each of the four study groups. To assess for possible dose-dependent effects, an analysis grouped by dosage of study drug was performed for the control and injected fibroid tissues. Analysis and data are shown according to their respective study group allocation. Fold change represents the average reduction in collagen content between control (set at 1.0) and injected samples. (**a**) Group 1, (**b**) Group 2 Dose 1, (**c**) Group 2 Dose 2, (**d**) Group 2 Dose 3. ^*^p <0.05, **p <0.01, and ***p-value <0.001(unpaired T-test)

Quantitative analysis of Masson’s trichrome-stained slides showed that all treated samples had a statistically significant reduction in collagen content compared to the controls (median reduction 39%, range 16-78%; *p* <0.001); (Fig. 3, Supplemental Figure 2). To assess for possible dose-dependent effects, a grouped analysis was performed to compare control and injected fibroid tissues according to the dose administered (Fig. 3, Table 3). There was a statistically significant reduction in the collagen content between control and treated fibroids in each study group. Using another approach, an additional analysis of comparing collagen content of treated fibroids to a pooled, normalized control group confirmed a notable reduction (median reduction 42.9%, range 12–64%, Supplemental Fig. 3).

**Table 3:** Changes in collagen content using a log linear mixed effects model for estimated ratio of intensity density of collagen by treatment and control group

Group	Intensity density^±^ of collagen ratio	*p*-value^*^	[95% conf. interval]		*p*-value for interaction
G1	0.514	<0.001	0.383	0.690	-
G2/D1	0.419	0.004	0.233	0.756	0.545
G2/D2	0.784	<0.001	0.732	0.840	0.006
G2/D3	0.533	<0.001	0.435	0.653	0.839

G1=Group 1; G2/D1=Group 2 Dose 1; G2/D2=Group 2 Dose 2; G2/D3=Group 2 Dose 3

± Intensity density is the sum of pixel values for collagen from ImageJ software analysis

Controls consisted of adjacent fibroids from the same subject. ^*^ Indicates a statistically significant change in collagen intensity density between treatment and control, *p*-value < 0.001

^**^Indicates a statistically significant difference in change in collagen intensity density between treatment and control for group2/D2 vs. group 1

**Fig. 4:**
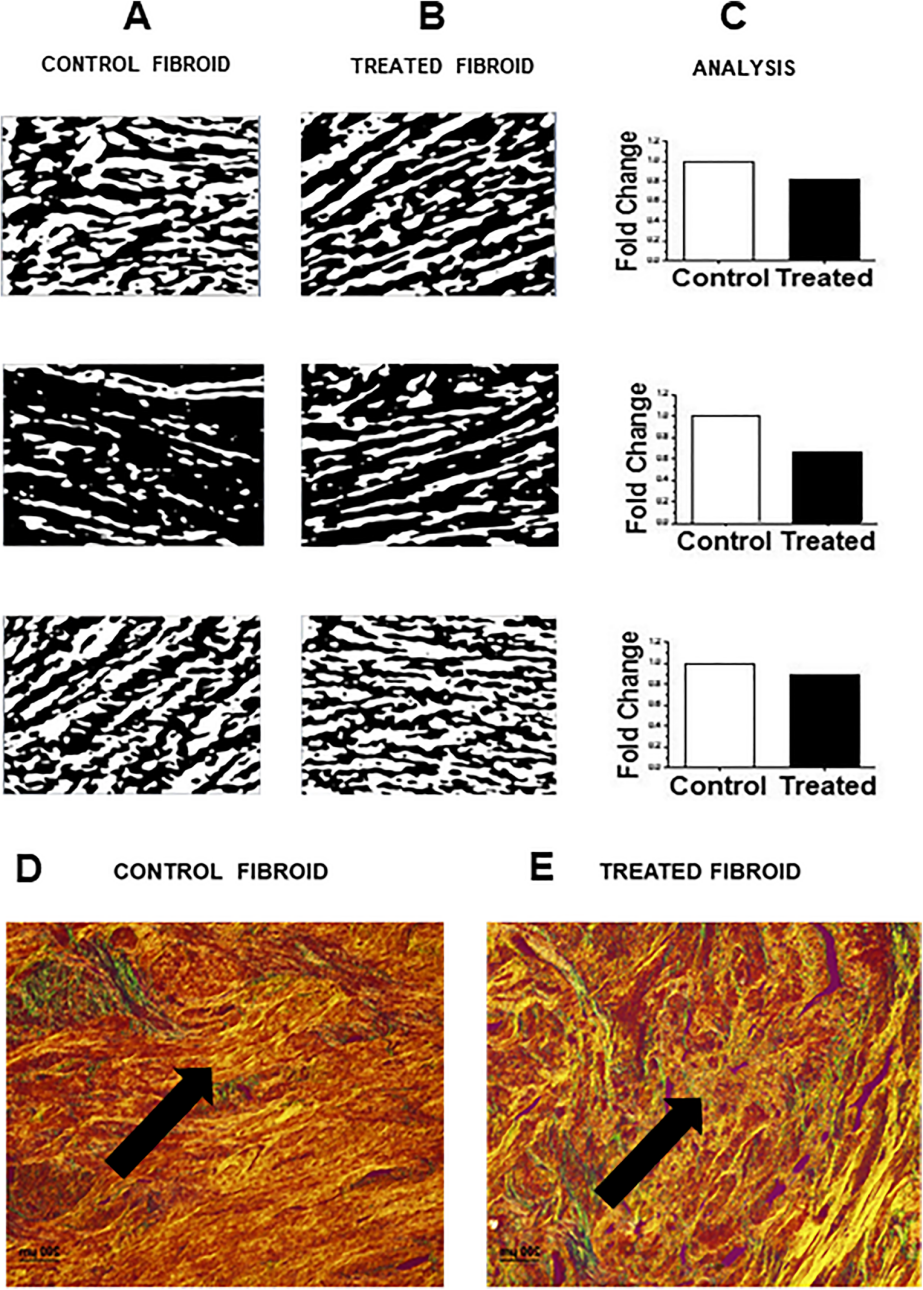
Quantification of collagen context in treated fibroids compared to adjacent fibroids. **a** and **b**, second harmonic generation imaging of the fibroid tissues. Collagen content appears black. **a** Control fibroids. **b** Treated fibroids. **c** Fold change in collagen distribution as measured by ImageJ software, change in density of collagen fiber distribution was measured in pixels (*n*=3). Representative micrograph of Picrosirius stained control (**d**) and treated (**e**) fibroid tissues under polarized light to visualize birefringence of collagen fibers, and the content was subjectively judged (*n*=12). Picrosirius staining shows collagen fibers as yellow in the control and yellow-green in the collagenase-treated tissues. Collagenase-treated tissues were less dense, and collagen fibers were shorter and oriented differently than in control tissues (black arrow), as shown on the right by the yellow-green staining (black arrow). One representative image is shown

SHG analysis showed that treated samples had an average of 21% (range 10–34%) reduction in distribution of collagen bundles compared to controls in each study group (Fig. 4). Picrosirius red-stained sections imaged under polarized light showed that collagen fibers in collagenase-treated tissues were less dense and shorter than in control tissues. Loss of collagen fibers was noted in treated fibroid tissues (Fig. 4). TUNEL assays did not detect an increase in apoptosis in all treated tissue sections compared to control. The tissue for analysis was obtained at the time of tissue harvesting post-hysterectomy. The control sections were obtained from matched fibroids from the same subject, and the treated fibroids sections were obtained from the injected fibroid from the subject (Supplemental Figure 4).

Though some samples suggested a reduction in size and reduced stiffness, we did not detect a significant change in size of the treated fibroids among treated fibroids by ultrasound or by assessment of the elasticity of uterine fibroids (data not shown), perhaps related to the small sample size, brief duration of exposure to collagen (Group 1), and the dosage used.

### Study Questionnaires

#### McGill Pain Questionnaire

In Group 1, no subject reported an increase in pain between baseline and 24–48 h post-injection, and two reported an average 5-point decrease in pain. In Group 2, only one of the nine subjects reported an increase in pain by one point between baseline and 4–8 days post-study drug injection (*p*=0.057), and no increase in pain was reported at 60–90 days post-study drug injection (pre-hysterectomy), (*p*=0.079). On average, there was a 14-point reduction in pain at 4–8 days for the other eight subjects in Group 2, and the trend continued for all subjects with an average 15-point reduction at 60–90 days from baseline.

#### Visual Analogue Scale

In Group 1, none of the subjects reported an increase in pain from baseline to 24–48 h post-study drug injection. In Group 2, seven out of nine subjects reported no increase in pain from baseline to 4–8 days post-study drug injection, and three out of nine subjects reported a mild increase in pain associated with fibroids at 60–90 days post-study drug injection. None of the changes were statistically significant.

#### Uterine Fibroid Symptom Health-Related Quality of Life Questionnaire

##### Symptom Severity Score UFS-QOL

In Group 1, 2 out of 3 subjects reported an increase in severity of symptoms associated with fibroids between baseline and 24–48 h post-study drug injection. In Group 2, 5 out of 9 subjects reported a mild decrease, 2 out of 9 reported a mild increase, and 2 subjects reported no change in symptom severity between baseline and 4–8 days post-study drug injection. Between baseline and 60–90 days post-study drug injection, five out of 9 subjects reported a decrease in symptom severity, and 4 out of 9 subjects reported a mild increase in symptom severity associated with fibroids.

##### Health-related Quality of Life Score UFS-QOL

In Group 1, all subjects reported an improvement in health-related quality of life between baseline and 24–48 h post-study drug injection. In Group 2, 4 out of 9 subjects reported a mild improvement, 3 out of 9 reported no change, and 2 out of 9 reported a decrease in quality of life from baseline to 4–8 days post-study drug injection. Four out of 9 subjects reported a mild improvement, 4 out of 9 reported a decrease, and one subject reported no change in quality of life associated with fibroids between baseline and 60–90 days post-study drug injection.

## Discussion

The results of this phase 1, open-label, and dose-escalation clinical trial found that injectable collagenase *Clostridium histolyticum* was safe and well-tolerated when injected directly into the center of a uterine leiomyoma. Despite the perceived difficulty of injecting uterine fibroids using a follicle aspiration needle, the study drug delivery transvaginally under ultrasound guidance was easy to achieve. This report expands upon ex vivo studies showing reduction of collagens in collagenase collagenase-treated tissues [11, 12].

When hemi-sectioned, all treated leiomyomas were soft to palpation and/or showed liquefaction in the center of the fibroid as compared to the periphery of the treated fibroid and the control fibroid from the same subject on gross examination. Histopathological examination using Masson’s trichrome stain revealed that treated leiomyomas had a statistically significant reduction in collagen content. Reduction in density and distribution of the collagen fibrils were observed using SHG analysis and Picrosirius staining. Thus, injectable collagenase *Clostridium histolyticum* significantly reduced the collagen content in the treated fibroid samples compared to controls at all treated doses. Though the observed reductions were slightly less for Group 2, Dose 2, the difference is most likely due to the small sample size of the groups, rather than other explanations such as operator-dependent effects. These findings supported the hypothesis that EN3835 was safe and well-tolerated when injected directly into uterine fibroids, thus satisfying the second primary outcome for the study. Further studies are needed to determine the optimal dosage and injection interval of EN3835 and whether the procedure might be performed as an outpatient procedure under local anesthesia, as is standard for treatment with EN3835 for Dupuytren’s and Peyronie’s diseases.

Eight out of nine subjects in Group 2 reported a notable reduction in fibroid-related pain at both the 4- to 8-day and 60- to 90-day post-injection time points, as evaluated by the McGill Pain Questionnaire. Since the study drug (EN3835) does not degrade the type IV collagen found in the basement membranes of the nerves and blood vessels, we hypothesize that the decrease in pain could be due to reduction in pressure resulting from the collagenase injection following study drug injection [18, 19]. All subjects in this phase 1 study received the study drug in the OR under heavy sedation. However, none of the subjects experienced significant levels of pain post-injection during recovery, and if pain relief was needed, acetaminophen provided sufficient relief. For the Saline only group, since the injections were done after induction of anesthesia and immediately before hysterectomy and there was no interval after injection of saline, their pain scores were not reported and collagen content was not assessed. The low number of adverse events, stable blood chemistry values and vital signs pre- and post-study drug injection, rapid clearance of the study drug within 4 h of injection, and minimal increase in the anti-AUX-I and anti-AUX-II antibodies along with the intact pseudo capsules of all injected fibroids post-study drug injection affirm the safety and tolerability of the study drug injection.

New drugs for medical management of uterine fibroids such as selective progesterone receptor modulators and oral GnRH antagonists are associated with a 50–60% reduction in fibroid size, but larger fibroids tend to persist and may continue to cause symptoms. EN3835 could be an effective combination agent to induce regression of fibroids during or following treatment with other medical therapies, to ensure better long-term outcomes in fibroid management. However, this study is an early study of safety and tolerability, and the clinical efficacy, dosage, number of required doses, and regimens need to be further evaluated. The details of future use of the injection, such as for mass reduction prior to surgery or repeated multiple injections rather than a single shot, are currently unclear. Possibly, patients interested in fibroid management with fertility preservation might be candidates for this therapy as it is minimally invasive and might aid in return to a normal uterine cavity more susceptible to conception. EN3835 and the drug delivery method described in this report, if future studies support its efficacy, may provide a new, non-hormonal treatment for uterine fibroids.

## Conclusions

Collagenase *Clostridium histolyticum* (EN3835) was safe and well tolerated when injected directly into uterine leiomyomas under ultrasound guidance. Treatment resulted in a significant reduction in collagen content in all treated fibroid samples. Future studies are warranted to determine the optimal dose and interval of injections to assess efficacy of multiple EN3835 injections directly into uterine leiomyomas.

## Supplementary Information

Suppl. Figure 1. Representative image of the fibroids injected in the Saline Only group. The black arrow points to the methylene blue injected into the center of the fibroid. (PDF 43 kb)

Suppl. Figure 2. Method of collagen quantification in treated and control tissues. Representative images of Masson’s Trichrome stained Control and Treated fibroid tissue collected at hysterectomy from 4 subjects at various doses of collagenase for Group1, 1.16 mg (Row A), and Group 2 Dose 1, (Row B), Dose 2, (Row C), & Dose 3, (Row D), with 1.68, 3.35, and 5.028 mg as the maximum doses respectively. The blue-green color represents the collagen in the colored images. The black & white images were generated using ImageJ software to quantify staining intensity and analyze collagen content. The black color represents the collagen. Collagen density was quantified using 9 grids with approximately 500.000 pixels. All treated samples showed a statistically significant reduction in collagen. Magnification is X 5. (PNG 2906 kb)

High resolution image (TIF 1349 kb)

Suppl. Figure 3. Alternative analysis of collagen quantification across treatment groups as compared to pooled control. To account for differences between collagen content among subjects, a normalized pooled control was used to compare study groups. Actual density (sum of pixel values) values are plotted in the Y axis and the study groups on the X axis, Controls (pooled data), G1: Group 1; G2D1: Group 2 Dose 1, G2D2: Group 2 Dose 2, G2D3: Group 2 Dose 3. On average there was a 42.9% (range 12.3-64.7%) reduction in collagen content between pooled control and study group samples. (PNG 135 kb)

High resolution image (TIF 53 kb)

Suppl. Figure 4. TUNEL Assay to detect apoptosis. No increase in apoptosis was identified in the treated fibroid samples collected post hysterectomy. Image A: Positive Control, Image B: Negative Control, Image C: Study Control, and Image D: Treated Sample (n=12, one representative image shown). (PDF 261 kb)

Suppl. Table A (DOCX 12 kb)
