# Supplementary figures and images for: A Phase I Clinical Trial to Assess Safety and Tolerability of Injectable Collagenase in Women with Symptomatic Uterine Fibroids
Source: Reprod Sci. 2021 Apr 29;28(9):2699–709. doi: 10.1007/s43032-021-00573-8 (PMC8346429; doi:10.1007/s43032-021-00573-8)

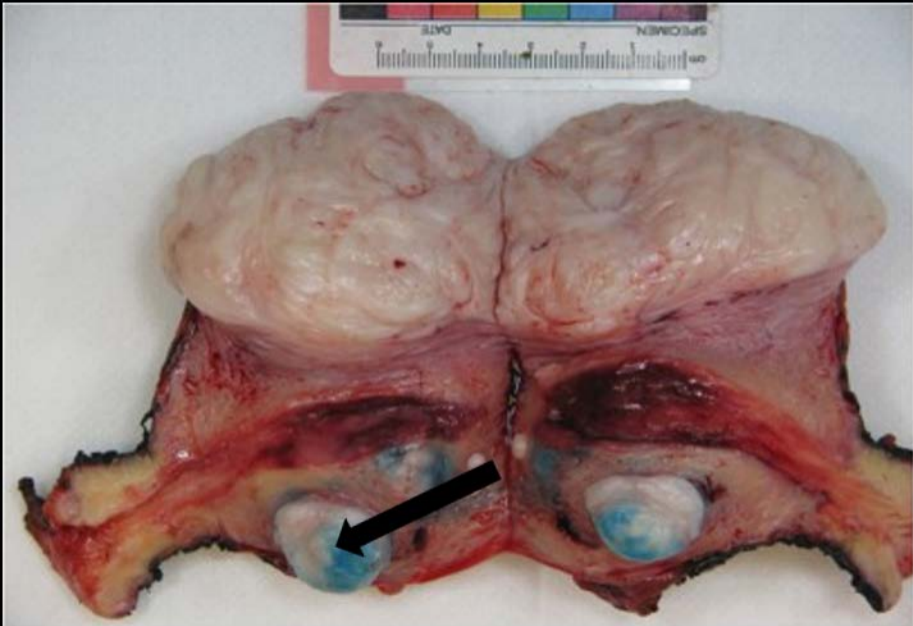

Supplement: Supplementary file 1 — Representative image of the fibroids injected in the Saline Only group. The black arrow points to the methylene blue injected into the center of the fibroid. (PDF 43 kb) [file 43032_2021_573_MOESM1_ESM.pdf]

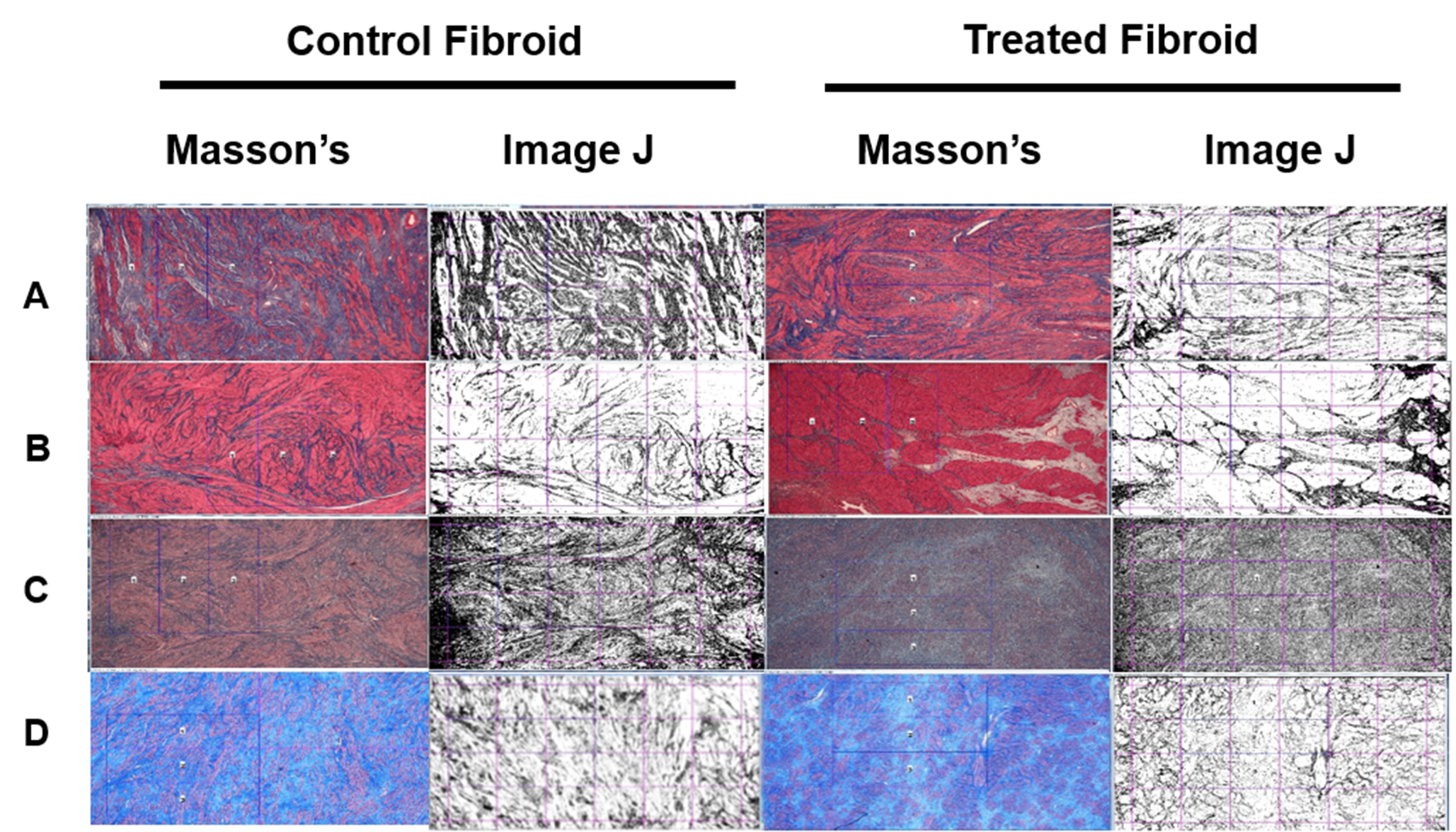

Supplement: Supplementary file 2 — Method of collagen quantification in treated and control tissues. Representative images of Masson’s Trichrome stained Control and Treated fibroid tissue collected at hysterectomy from 4 subjects at various doses of collagenase for Group1, 1.16 mg (Row A), and Group 2 Dose 1, (Row B), Dose 2, (Row C), & Dose 3, (Row D), with 1.68, 3.35, and 5.028 mg as the maximum doses respectively. The blue-green color represents the collagen in the colored images. The black & white images were generated using ImageJ software to quantify staining intensity and analyze collagen content. The black color represents the collagen. Collagen density was quantified using 9 grids with approximately 500.000 pixels. All treated samples showed a statistically significant reduction in collagen. Magnification is X 5. (PNG 2906 kb) [file 43032_2021_573_Fig5_ESM.png]

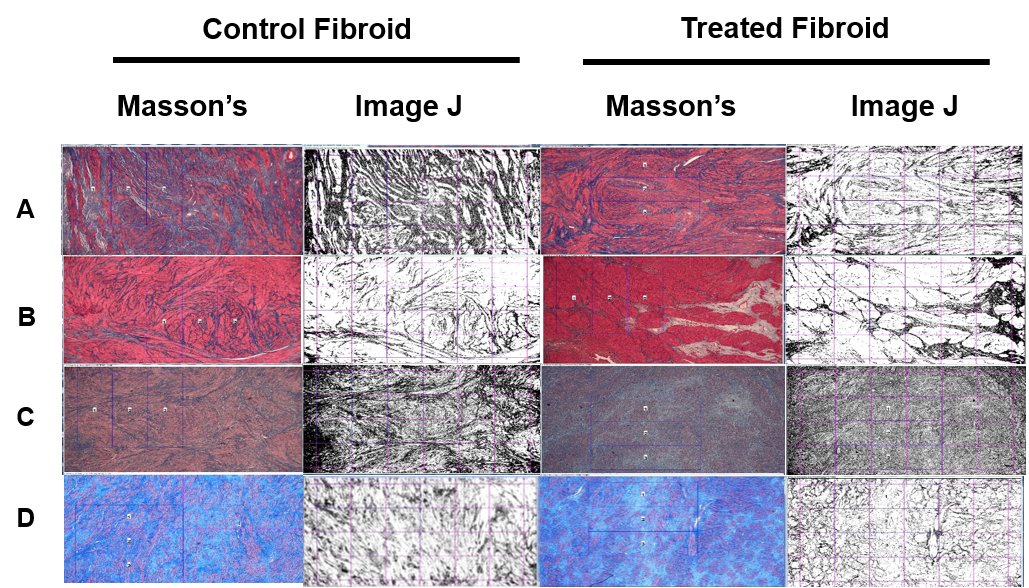

Supplement: Supplementary file 3 — High resolution image (TIF 1349 kb) [file 43032_2021_573_MOESM2_ESM.tif]

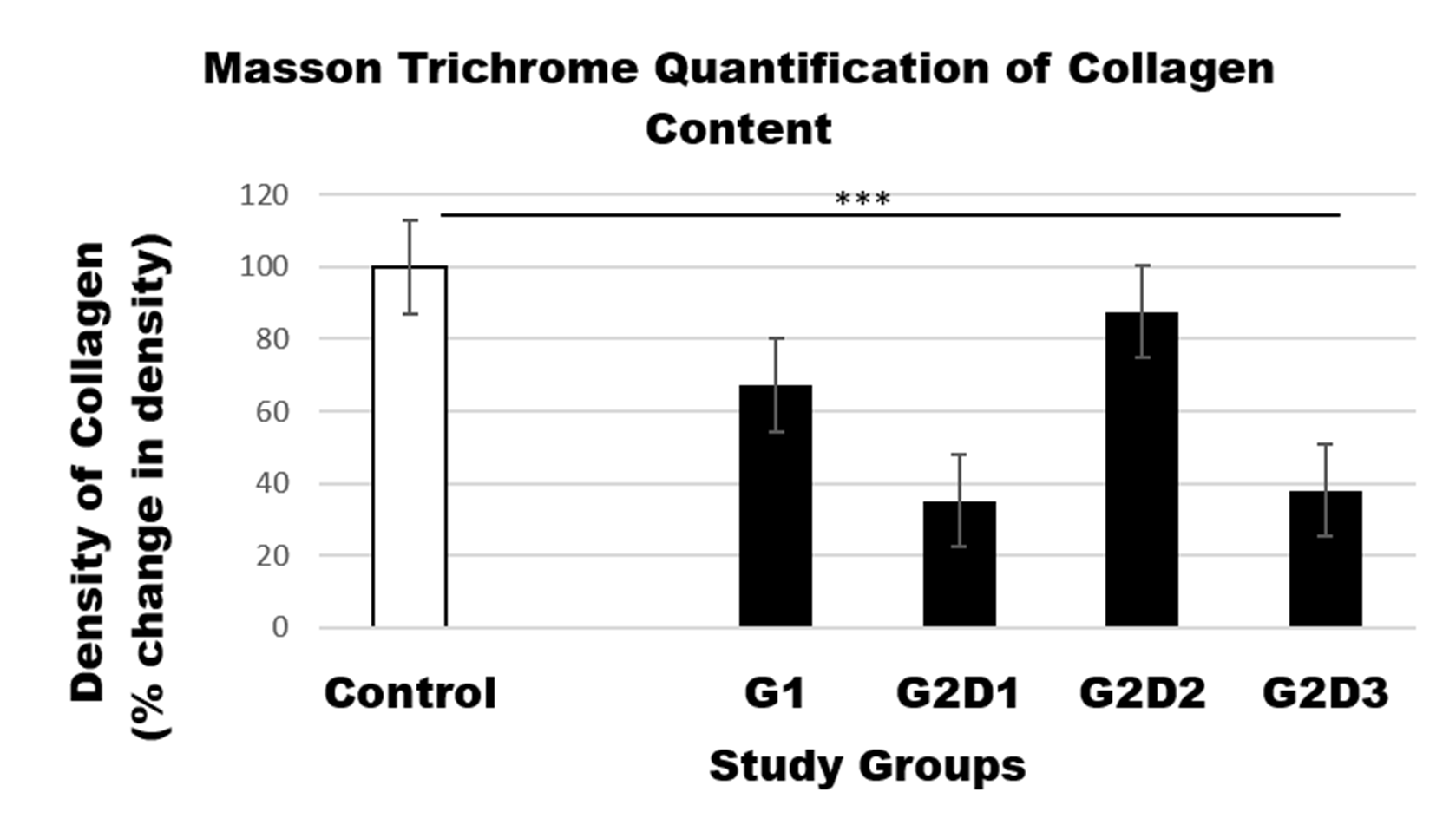

Supplement: Supplementary file 4 — Alternative analysis of collagen quantification across treatment groups as compared to pooled control. To account for differences between collagen content among subjects, a normalized pooled control was used to compare study groups. Actual density (sum of pixel values) values are plotted in the Y axis and the study groups on the X axis, Controls (pooled data), G1: Group 1; G2D1: Group 2 Dose 1, G2D2: Group 2 Dose 2, G2D3: Group 2 Dose 3. On average there was a 42.9% (range 12.3-64.7%) reduction in collagen content between pooled control and study group samples. (PNG 135 kb) [file 43032_2021_573_Fig6_ESM.png]

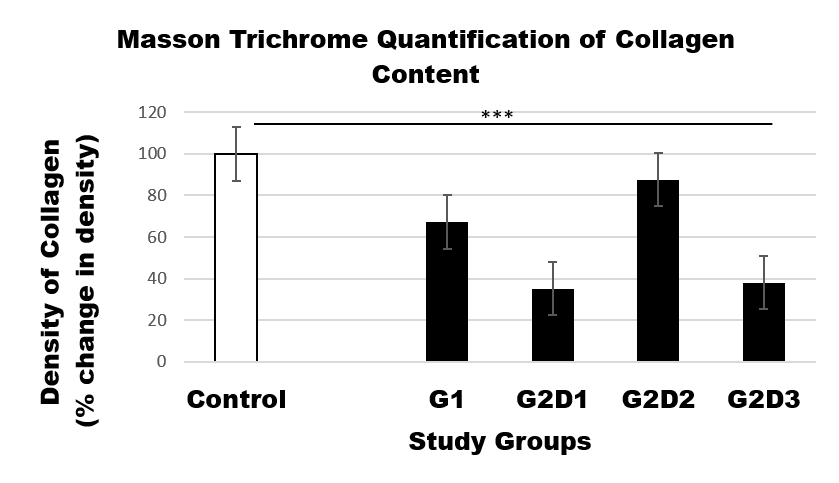

Supplement: Supplementary file 5 — High resolution image (TIF 53 kb) [file 43032_2021_573_MOESM3_ESM.tif]

**POSITIVE  
CONTROL**

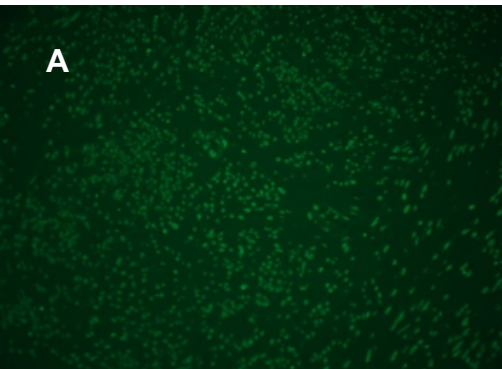

**NEGATIVE  
CONTROL**

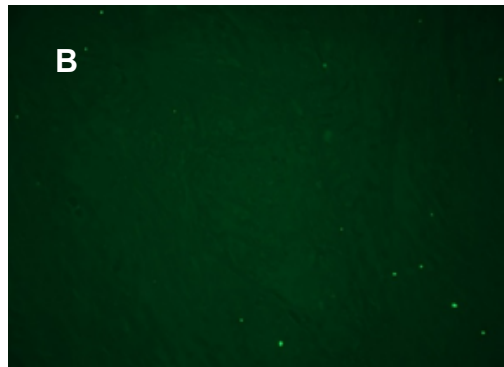

**STUDY  
CONTROL**

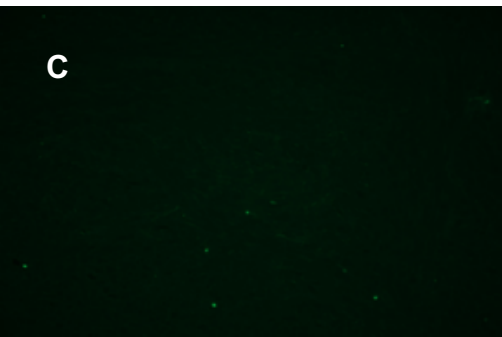

**STUDY  
TREATED**

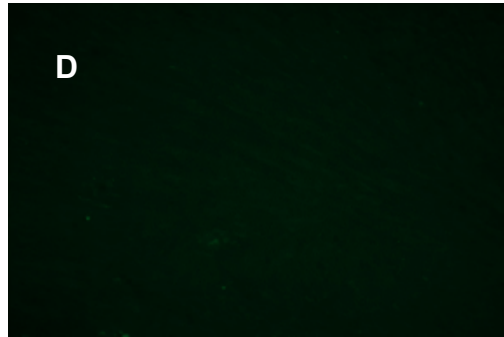

Supplement: Supplementary file 6 — TUNEL Assay to detect apoptosis. No increase in apoptosis was identified in the treated fibroid samples collected post hysterectomy. Image A: Positive Control, Image B: Negative Control, Image C: Study Control, and Image D: Treated Sample (n=12, one representative image shown). (PDF 261 kb) [file 43032_2021_573_MOESM4_ESM.pdf]
