# Supplementary material for: A Phase I Clinical Trial to Assess Safety and Tolerability of Injectable Collagenase in Women with Symptomatic Uterine Fibroids
Source: Reprod Sci. 2021 Apr 29;28(9):2699–709. doi: 10.1007/s43032-021-00573-8 (PMC8346429; doi:10.1007/s43032-021-00573-8)
Supplement: Supplementary file 7 — (DOCX 12 kb) [file 43032_2021_573_MOESM5_ESM.docx]

| **Group** | **Subject ID** | **Total number of fibroids** | **Largest diameter of injected fibroid(in cm)** | Study drug dosage (mg) |
| --- | --- | --- | --- | --- |
| **Saline Only Group** | FIB_002_6589 | 3 major | 3.2, 4.6 | 0 |
|  | FIB_003_0594 | 10, 5 major, 5 minor | 3.45 | 0 |
|  | FIB_004_9539 | 1 major, 4 minor | 6.85, 4.34 | 0 |
| **Group 1** | FIB_006_1474 | 5 major+ multiple minor | 4.01 | 1.16 |
|  | FIB_007_4898 | 5 major+ multiple minor | 5.2 | 1.16 |
|  | FIB_009_3785 | 5 major | 4.2 | 1.16 |
| **Group 2 Dose 1** | FIB_010_6378 | 2 major, 4 minor | 8 | 1.68 |
|  | FIB_011_2090 | 3 major | 3.1 | 0.71 |
|  | FIB_012_0836 | 5 major | 4.5 | 1.68 |
| **Group 2 Dose 2** | FIB_013_8676 | >15, 5 major, multiple minor | 4.73 | 3.35 |
|  | FIB_014_9766 | 5, 2 major, 2 minor | 3.34 | 1.41 |
|  | FIB_015_3113 | 3 major, 1 minor | 4.15 | 3.35 |
| **Group 2 Dose 3** | FIB_017_5270 | 4 major, multiple minor | 4.41 | 5.02 |
|  | FIB_018_9223 | 2 major | 3.02 | 2.83 |
|  | FIB_019_3634 | 2 major | 6.16 | 5.02 |

Supplementary Table A: Fibroid size and study drug dosage

*Largest diameter > 3cm- major, minor < 3 cm
